# Impact of the South Korean government’s medical school expansion announcement on pediatric emergency department visits

**DOI:** 10.1186/s12873-025-01189-w

**Published:** 2025-03-05

**Authors:** Arum Choi, Beom Joon Kim, Jooyoung Lee, Sukil Kim, Woori Bae

**Affiliations:** 1 Department of Radiology, College of Medicine, The Catholic University of Korea, Seoul, Republic of Korea; 2 Department of Pediatrics, Eunpyeong St. Mary’s Hospital, College of Medicine, The Catholic University of Korea, Seoul, Republic of Korea; 3 Department of Preventive Medicine and Public Health, College of Medicine, The Catholic University of Korea, Seoul, Republic of Korea; 4 Department of Emergency Medicine, Seoul St. Mary’s Hospital, College of Medicine, The Catholic University of Korea, Seoul, Republic of Korea

**Keywords:** Emergency Department visits, Healthcare policy, Pediatric emergency department, Residents resignation, Strikes

## Abstract

**Background:**

In February 2024, the South Korean government announced a 67% increase in medical school admissions (2,000 more students), leading to the resignation of approximately 10,000 residents from major university hospitals. This study investigated the impact of these resignations on pediatric emergency department (PED) visits at a major tertiary hospital in Korea.

**Methods:**

We conducted a retrospective observational study analyzing PED visits under 15 years old at a tertiary hospital from January 2019 to May 12, 2024. After excluding cases with missing diagnostic codes or disposition records, we analyzed visits during the 12-week period from February 19 to May 12 across different years (2019–2024). We used segmented regression of Interrupted Time Series (ITS) analysis to evaluate the impact of three key events: the COVID-19 onset, lifting of mask-wearing mandates, and residents’ resignation, adjusting for seasonal variations and autocorrelation.

**Results:**

Among 11,574 analyzed cases, weekly PED visits decreased significantly after residents’ resignation (133.6 ± 22.4) compared to pre-COVID-19 (246.3 ± 45.2) and post-COVID-19 (263.7 ± 61.2) periods. The proportion of KTAS 3 cases increased to 67.2% during the resignation period compared to pre-COVID-19 (48.9%). ITS analysis revealed significant immediate changes in weekly visits: COVID-19 (-157.81 visits, 95% CI: -202.04 to -113.58), mask mandate removal (48.26 visits, 95% CI: 3.21 to 93.32), and residents’ resignation (-77.82 visits, 95% CI: -134.85 to -20.80). Notably, the proportion of infectious diseases increased (36.9% vs. 18.6% pre-COVID-19), while respiratory diseases decreased (20.1% vs. 33.6% pre-COVID-19).

**Conclusion:**

A substantial reduction in both absolute and relative weekly patient visits was observed following the start of the nationwide resident strike at our pediatric emergency department. Additional studies are needed to better understand how this affected pediatric emergency care delivery and access.

## Background

In February 2024, the South Korean government announced an increase of 2,000 medical school admissions starting from 2025 to address the doctor shortage [1]. It represents a 67% increase in the current medical school capacity of 3,058. In response to the announcement, approximately 10,000 residents at major university hospitals submitted letters of resignation and left hospitals as of February 19, 2024 [2, 3].

At major university hospitals, which train more residents than the other hospitals, dependency on residents was reported to be 30–40%, compared to approximately 10% reported in major hospitals in Japan or the United States [4]. Residents continuously monitor the patient’s condition, decide on admission, and transfer patients to other specialties as needed. Residents are essential personnel who significantly contribute to PED operations through their continuous presence and immediate response to patient care [5].

In August 2020, more than 80% of the residents went on strike for a month, demanding the withdrawal of government plans to increase medical students’ numbers [6]. A study on the impact of this strike found that while there was no impact on the mortality rate of patients visiting the emergency departments (EDs), there was an increase in the number of patients admitted and length of stay during the period [7].

The purpose of this study is to investigate the impact of residents’ resignation caused by the announcement of expanding medical school admissions on visits to PED.

## Methods

### Study design

We conducted a retrospective observational study analyzing the impact of residents’ resignation on PED visits at a major university hospital in South Korea from January 1, 2019, and May 12, 2024.

### Study setting

The study was conducted in a tertiary hospital with approximately 1,400 beds one of the five major university hospitals in South Korea. The hospital’s PED serves approximately 10,000 patients annually. Pediatric residents undergo a four-year training program, with 16 total residents (four per year). Three residents simultaneously rotate through the PED while also covering the pediatric ward.

### Participants

**Fig. 1:**
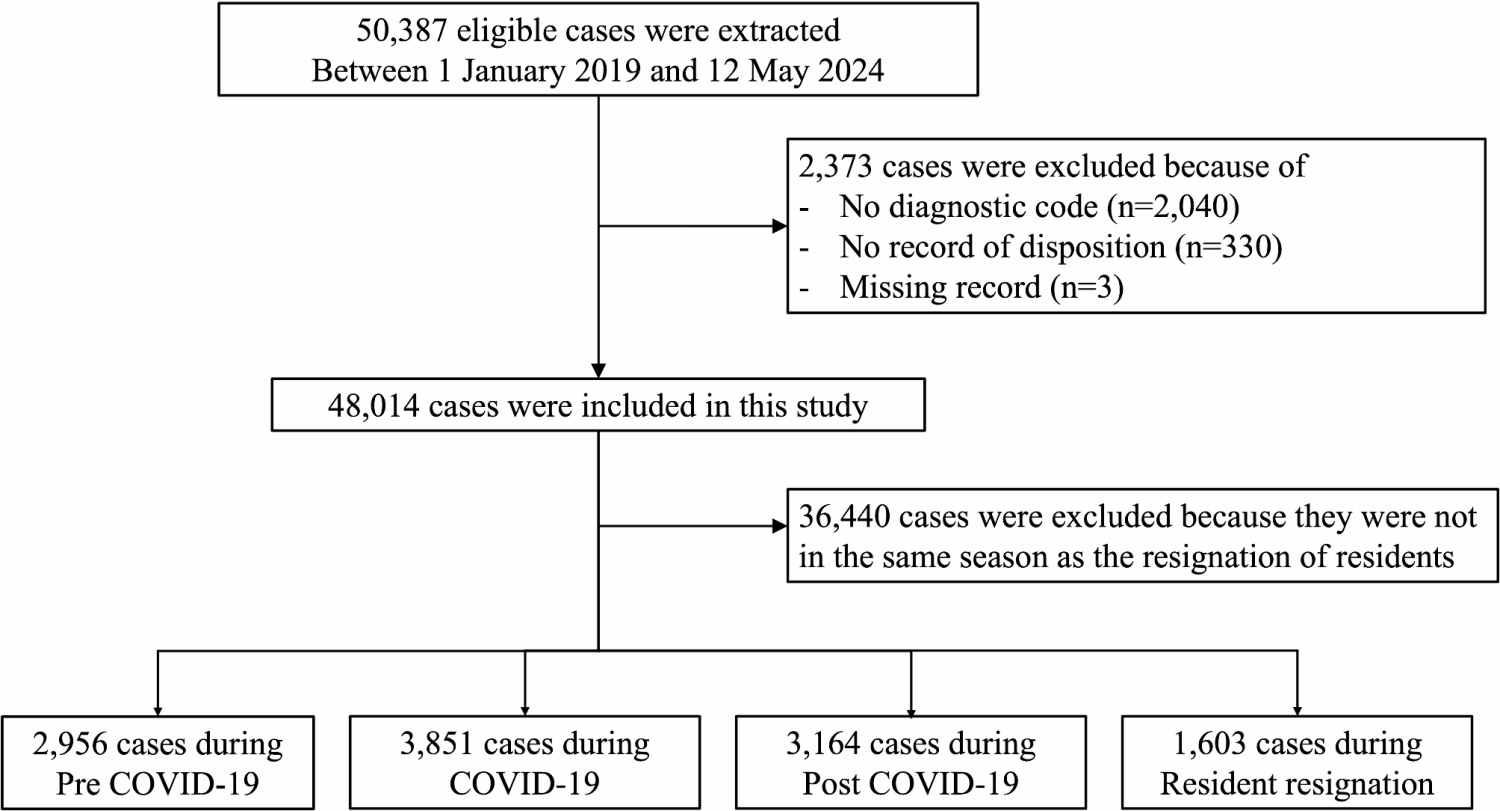
Flowchart of the study population

The study included patients aged less than 15 years who visited the PED. To enable direct comparison and control for seasonal variations, we analyzed visits during the 12-week period from February 19 to May 12 of each year (2019–2024), as the residents’ resignation began on February 19, 2024. From the initial 50,387 cases recorded between January 1, 2019, and May 12, 2024, we excluded 2,373 cases due to missing diagnostic codes, disposition records, or recording errors (*n* = 48,014). After excluding 36,440 cases that fell outside our defined study period (February 19 to May 12), the final analysis included 11,574 cases across four periods: pre-COVID-19 (*n* = 2,956), during COVID-19 (*n* = 3,851), post-COVID-19 (*n* = 3,164), and post-resident resignation (*n* = 1,603) (Fig. 1).

### Variables

We extracted the following variables from electronic medical records: age, sex, triage acuity, diagnoses, mode of arrival, PED length of stay, and disposition. Triage acuity was measured using the Korean Triage and Acuity Scale (KTAS) [8], which ranges from 1 (critical) to 5 (non-urgent), with scores of 1–3 classified as high-acuity cases. Diagnoses were made using the diagnostic codes of the Korean Standard Classification of Diseases 7th revision (KCD-7), based on the International Classification of Diseases-10 (ICD-10) of the World Health Organization [9]. Diagnoses were categorized into nine groups based on the first letter of their ICD-10 codes: respiratory (J codes), gastrointestinal (K codes), haemato-oncologic (C and D codes), neurologic (G codes), trauma (S and T codes), infectious (A and B codes), urogenital (N codes), cardiac (I codes), and others (remaining codes). For patients with multiple diagnoses, only the primary discharge diagnosis was used for categorization.

### Outcomes

We compared and analyzed the number of PED visits per week and the differences in the diagnoses of patients visiting the PED during the study period.

### Statistical analysis

We used one-way ANOVA followed by Tukey’s post-hoc test to compare weekly visit means between periods. For non-normally distributed data (PED length of stay), we used the Kruskal-Wallis test with Dunn’s post-hoc test for multiple comparisons. Categorical variables (age groups, sex, KTAS levels, arrival mode, disposition, and diagnostic categories) were analyzed using chi-square tests. When the overall chi-square test was significant, we conducted post-hoc analysis using Bonferroni-adjusted standardized residuals to identify specific between-group differences.

We used segmented regression of Interrupted Time Series (ITS) analysis to evaluate the impact of three key events on PED visits: the onset of the COVID-19 pandemic (January 27, 2020), the lifting of mask-wearing mandates (January 30, 2023), and the resignation of residents (February 19, 2024). The model incorporated seasonal variables avoiding potential distortions due to the pandemic and was constructed using generalized least squares (GLS) to account for autocorrelation.

The final regression model is:

$$\eqalign{Yt\:& =\:\beta\:0\:+\:\beta\:1time\:+\:\beta\:2covid19 \cr &+\:\beta\:3time\_after\_covid19\:+\:\beta\:4mask\_off \cr & +\:\beta\:5time\_after\_mask\_off\:+\:\beta\:6resident\_resign \cr & +\:\beta\:7time\_after\_resident\_resign\:+\:\beta\:8sin(2\pi\:week/52) \cr & +\:\beta\:9cos(2\pi\:*week/52)\:+\:\epsilon\:\_t }$$

Where ε_t represents the error term with an ARMA (1,0) correlation structure.

All analyses were performed using R version 4.4.0 (R Foundation for Statistical Computing, Vienna, Austria), with the probability level for significance set at a P-value < 0.05.

## Results

### Demographics of PED patients

The characteristics of patients across different time periods—pre-COVID-19, during COVID-19 (2020, 2021, and 2022), post-COVID-19, and during residents’ resignation —are shown in Table 1. One-way ANOVA with Tukey’s post-hoc test revealed that the average number of weekly visits after residents’ resignation (133.6 ± 22.4) was significantly lower than pre-COVID-19 (246.3 ± 45.2) and post-COVID-19 periods (263.7 ± 61.2) (both *P* < 0.001). Among age groups, children aged 3–7 years comprised the largest proportion during the pre-COVID-19 (31.9%) and post-COVID-19 (35.6%) periods, while the proportion of infants aged 0–12 months significantly increased during the residents’ resignation period (14.4% vs. 9.9% pre-COVID-19, *P* < 0.05). After resignation, the proportion of KTAS 3 cases increased significantly to 67.2% compared to pre-COVID-19 (48.9%) and during COVID-19 periods (35.3–52.8%) (*P* < 0.001), while KTAS 4 cases decreased significantly (24.8% vs. 44.8% pre-COVID-19, *P* < 0.001). The median PED length of stay was significantly shorter during the resignation period (106.0 min, IQR 45.0-187.0) compared to other periods (124.0–134.0 min) (*P* < 0.001). After resignation, while the proportion of self-referred patients decreased to 81.6%, the proportion of clinic referrals reached its highest at 16.9% (*P* < 0.001).

**Table 1:** Characteristics of pediatric emergency department patients, N (%)

Variables/Category		Pre COVID-19	COVID-19 pandemic			Post COVID-19	After residents’ resignation	*P*
		Feb 19– May 12, 2019	Feb 19– May 12, 2020	Feb 19– May 12, 2021	Feb 19– May 12, 2022	Feb 19– May 12, 2023	Feb 19– May 12, 2024	
Total visit		2,956	1,003	1,470	1,378	3,164	1,603	
Weekly visit, mean ± SD †		246.3 ± 45.2 ^a^	83.6 ± 12.5 ^c^	122.5 ± 18.9 ^b^	114.8 ± 17.9 ^b^	263.7 ± 61.2 ^a^	133.6 ± 22.4 ^b^	< 0.001^*^
Age^*^	0-12months	293 (9.9%) ^a^	130 (13.0%) ^b^	162 (11.0%) ^ab^	199 (14.4%) ^b^	354 (11.2%) ^ab^	231 (14.4%) ^b^	< 0.001
	1-3years	852 (28.8%) ^a^	279 (27.8%) ^a^	430 (29.3%) ^a^	326 (23.7%) ^b^	913 (28.9%) ^a^	450 (28.1%) ^a^	
	3-7years	942 (31.9%) ^b^	298 (29.7%) ^a^	392 (26.7%) ^b^	376 (27.3%) ^b^	1126 (35.6%) ^a^	476 (29.7%) ^a^	
	7-14years	869 (29.4%) ^a^	296 (29.5%) ^a^	486 (33.1%) ^a^	477 (34.6%) ^a^	771 (24.4%) ^b^	446 (27.8%) ^ab^	
Female		1,323 (44.8%) ^a^	408 (40.7%) ^b^	627 (42.7%) ^ab^	553 (40.1%) ^b^	1319 (41.7%) ^b^	685 (42.7%) ^ab^	0.041
KTAS level^*^	Level 1	3 (0.1%)	4 (0.4%)	1 (0.1%)	1 (0.1%)	2 (0.1%)	2 (0.1%)	< 0.001
	Level 2	73 (2.5%) ^a^	31 (3.1%) ^ab^	62 (4.2%)	68 (4.9%) ^b^	132 (4.2%) ^b^	81 (5.1%) ^b^	
	Level 3	1,445 (48.9%) ^a^	354 (35.3%) ^b^	746 (50.7%)	728 (52.8%) ^a^	1,992 (63.0%) ^c^	1,077 (67.2%) ^c^	
	Level 4	1,323 (44.8%) ^a^	579 (57.7%) ^b^	627 (42.7%)	556 (40.3%)	963 (30.4%) ^c^	397 (24.8%) ^c^	
	Level 5	112 (3.8%)	35 (3.5%)	34 (2.3%)	25 (1.8%)	75 (2.4%)	46 (2.9%)	
PED Length of stay, median (IQR) §		124.0 (71.0-255.0) ^a^	103.0 (54.0-239.5) ^b^	125.0 (60.0-300.0)	130.0 (63.0-299.0) ^a^	133.5 (61.0-231.0) ^a^	106.0 (45.0-186.5) ^b^	< 0.001
Arrival mode^*^	Self-referred	2,600 (88.0%) ^a^	835 (83.3%) ^b^	1,201 (81.7%)	1,153 (83.7%) ^b^	2,687 (84.9%) ^b^	1,308 (81.6%) ^b^	< 0.001
	Referred from clinic	278 (9.4%) ^a^	114 (11.4%) ^ab^	174 (11.8%)	166 (12.0%) ^b^	371 (11.7%) ^b^	271 (16.9%) ^c^	
	Outpatient department	78 (2.6%) ^a^	54 (5.4%) ^b^	95 (6.5%)	59 (4.3%) ^ab^	106 (3.4%) ^a^	24 (1.5%) ^a^	
Disposition^*^	Discharge	2,624 (88.8%) ^a^	856 (85.3%) ^ab^	1,221 (83.1%)	1,140 (82.7%) ^b^	2,758 (87.2%) ^a^	1,381 (86.2%) ^ab^	< 0.001
	Hospitalization							
	Ward	327 (11.1%) ^a^	138 (13.8%) ^ab^	244 (16.6%)	232 (16.8%) ^b^	399 (12.6%) ^a^	219 (13.7%) ^ab^	
	PICU	5 (0.2%)	8 (0.8%)	5 (0.3%)	6 (0.4%)	7 (0.2%)	3 (0.2%)	
	Mortality	0 (0.0%)	1 (0.1%)	0 (0.0%)	0 (0.0%)	0 (0.0%)	0 (0.0%)	

All values are frequencies (%) except where otherwise indicated.

† One-way ANOVA with Tukey’s post-hoc test

§ Kruskal-Wallis test with Dunn’s post-hoc test

Chi-square test with Bonferroni-adjusted standardized residuals

Different superscript letters (a, b, c) indicate significant differences between groups (*P* < 0.05)

PED, Pediatric Emergency Department; KTAS, Korean Triage and Acuity Scale; PICU, Pediatric Intensive Care Unit; IQR, Interquartile Range; SD, Standard Deviation

### Interrupted time-series analysis of weekly PED visits

**Fig. 2:**
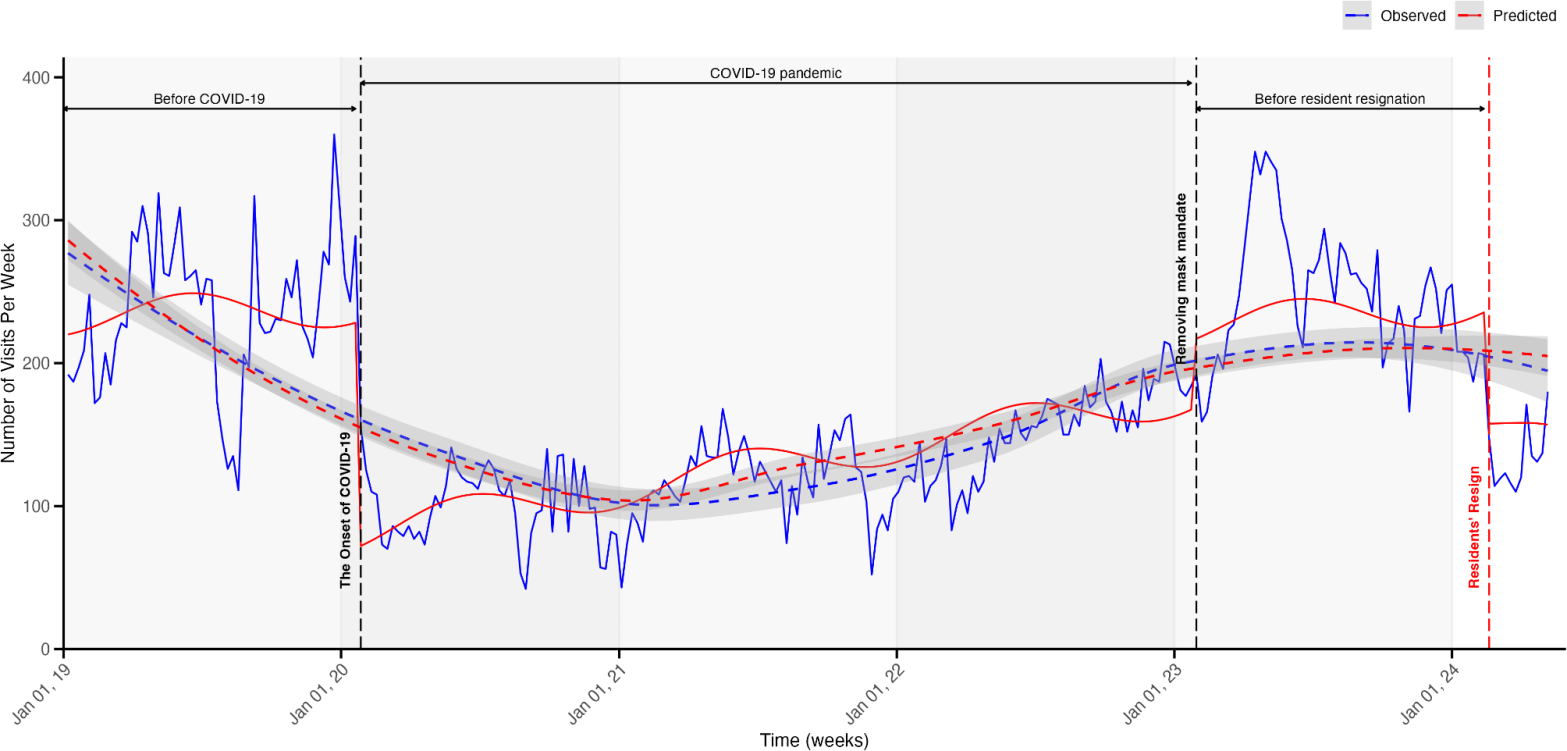
Interrupted Time Series Analysis of weekly PED visit from January 1, 2019 to May 12, 2024. The figure illustrates the trends in weekly PED visits during the study period, highlighting the impact of three key events: the onset of the COVID-19 pandemic, the lifting of the mask mandate, and residents’ resignation. The blue line represents observed PED visits, while the red line shows model-fitted values obtained using a generalized least squares model. Vertical dashed lines indicate: (1) the onset of the COVID-19 pandemic (January 27, 2020), (2) the lifting of the mask mandate (January 30, 2023), and (3) residents’ resignation (February 19, 2024). Confidence intervals around the fitted values demonstrate changes in trends over time. PED, pediatric emergency department; COVID-19, coronavirus disease 2019

The result of ITS analysis revealed significant changes in PED visits associated with key events. The onset of COVID-19 was associated with a decrease of 157.81 visits per week (95% CI: -202.04 to -113.58). The removal of the mask mandate led to a increase of 48.26 visits per week (95% CI: 3.21 to 93.32). The resident resignation resulted in a decrease of 77.82 visits per week (95% CI: -134.85 to -20.80). However, none of the interventions showed significant changes in the long-term trends of weekly visits (Fig. 2; Table 2).

**Table 2:** Interrupted time-series analysis of weekly PED visits

Parameter	Estimate	Std. Error	t value	Confidence interval	*P* ^*^	
Initial level	232.33	25.21	9.22	182.92 to 281.74	< 0.001	
Time (weeks)	0.12	0.70	0.17	-1.25 to 1.49	0.86	
COVID-19 pandemic	-157.81	22.57	-6.99	-202.04 to -113.58	< 0.001	
During COVID-19 pandemic	0.49	0.75	0.66	-0.97 to 1.95	0.51	
Lifting mandatory mask wearing	48.26	22.99	2.10	3.21 to 93.32	< 0.05	
After lifting mandatory mask wearing	-0.32	0.75	-0.43	-1.8 to 1.16	0.67	
Residents’ resignation	-77.82	29.09	-2.67	-134.85 to -20.8	< 0.05	
After residents’ resignation	-1.79	4.92	-0.36	-11.43 to 7.86	0.72	

* *p*-values from the Generalized Least Squares regression analysis.

### Diagnostic categories of PED visitors

We observed significant differences in the distribution of diagnostic categories between periods (all *Ps* < 0.001). In the resignation period, infectious diseases showed the highest proportion (591/1,603, 36.9%) compared to pre-COVID-19 (551/2,956, 18.6%). The proportion of respiratory diseases decreased significantly (323/1,603, 20.1% in the resignation period vs. 993/2,956, 33.6% in pre-COVID-19), as did trauma cases (303/1,603, 18.9% in the resignation period vs. 806/2,956, 27.3% in pre-COVD-19). Several other diagnostic categories showed notable changes: neurological (45/1,603, 2.8% in the resignation period vs. 34/2,956, 1.2% in pre-COVID-19) haemato-oncologic (61/1,603, 3.8% in the resignation period vs. 109/2,956, 3.7% in pre-COVID-19), and urogenital diseases (44/1,603, 2.7% in the resignation period vs. 53/2,956, 1.8% in pre-COVID-19). The detailed changes in diagnostic categories are shown in Table 3.

**Table 3:** Diagnostic categories of PED attendances, N (%)

Variables/Category		Pre COVID-19	COVID-19 pandemic			Post COVID-19	After residents’ resignation	*P* ^*^
		Feb 19– May 12, 2019	Feb 19– May 12, 2020	Feb 19– May 12, 2021	Feb 19– May 12, 2022	Feb 19– May 12, 2023	Feb 19– May 12, 2024	
Diagnoses	Respiratory	993 (33.6%) ^a^	152 (15.2%) ^b^	217 (14.8%) ^b^	181 (13.1%) ^b^	1000 (31.6%) ^a^	323 (20.1%) ^c^	< 0.001
	Gastrointestinal	98 (3.3%) ^ab^	50 (5.0%) ^c^	60 (4.1%) ^ab^	68 (4.9%) ^c^	74 (2.3%) ^a^	40 (2.5%) ^ab^	
	Hemato-oncologic	109 (3.7%) ^a^	38 (3.8%) ^ab^	53 (3.6%) ^a^	79 (5.7%) ^b^	89 (2.8%) ^a^	61 (3.8%) ^ab^	
	Neurological	34 (1.2%) ^a^	20 (2.0%) ^ab^	23 (1.6%) ^a^	23 (1.7%) ^a^	36 (1.1%) ^a^	45 (2.8%) ^b^	
	Trauma	806 (27.3%) ^a^	439 (43.8%) ^c^	562 (38.2%) ^b^	434 (31.5%) ^a^	593 (18.7%) ^d^	303 (18.9%) ^d^	
	Infectious	551 (18.6%) ^a^	134 (13.4%) ^b^	287 (19.5%) ^a^	306 (22.2%) ^a^	976 (30.8%) ^c^	591 (36.9%) ^c^	
	Uro-genital	53 (1.8%) ^ab^	38 (3.8%) ^c^	30 (2.0%) ^ab^	35 (2.5%) ^ab^	37 (1.2%) ^a^	44 (2.7%) ^bc^	
	Cardiac	3 (0.1%) ^a^	7 (0.7%) ^b^	13 (0.9%) ^b^	9 (0.7%) ^b^	7 (0.2%) ^a^	3 (0.2%) ^a^	
	Others	309 (10.5%) ^a^	125 (12.5%) ^ab^	225 (15.3%) ^b^	243 (17.6%) ^b^	352 (11.1%) ^a^	193 (12.0%) ^ab^	

All values are frequencies (%) except where otherwise indicated.

Chi-square test with Bonferroni-adjusted post-hoc analysis Different superscript letters (a, b, c, d) within the same row indicate significant differences between groups (*P* < 0.05 after Bonferroni correction) P-values are from chi-square test

PED, Pediatric Emergency Department

## Discussion

The results highlighted significant changes in PED visits after the residents’ resignation. During this period, the average weekly visits to the PED were notably lower than in pre-COVID-19 and post-COVID-19 periods. The percentage of high-acuity cases was higher after the residents’ resignation compared to other periods. Furthermore, the percentage of self-referred patients was the lowest, while the percentage of patients referred from the clinic was the highest after the resident resignation. Additionally, there were shifts in diagnostic categories, with a decrease in respiratory diseases and an increase in neurological diseases compared to the pre-COVID-19 and post-COVID-19 periods.

### Direct impact of medical school expansion policy and residents’ resignation

On February 16, 2024, residents began submitting resignations, and as of February 19, 6,415 residents, or 55% of them, submitted their resignations, and the number of residents who submitted resignations by February 23rd was 10,34, which is about 80% of all residents. As a result, various media broadcast that patients are restricted from using EDs, and even some patients who are transported by ambulance cannot be accommodated in EDs.

Residents’ resignation had a direct and profound impact on PED visits. The number of patients visiting the PED after the residents’ resignation decreased 49% compared with the same period in previous years. A previous study reported that during the 2016 junior doctors’ strike in the UK, ED visits fell by 5–13% [10]. The proportion of junior doctors in the hospitals that participated in the strike was approximately 50% higher; however, the strike lasted only a few days, which may have had less of an impact on the EDs. In contrast, ongoing residents’ resignation in South Korea for months have significantly impacted PED patient visits. The extended absence of residents responsible for the initial pediatric care, patients and caregivers may have refrained from visiting the PED because they were unsure whether the PED was functioning at the university hospital. During the COVID-19 pandemic, the number of patients visiting the PED decreased significantly as the mask mandate was implemented, and during the same period last year, the number of patients visiting the PED increased significantly again as the mask mandate was lifted. Since then, despite the lack of government-level policies on mask mandate and infectious disease management, the rapid decline in the number of patients with respiratory diseases visiting the PED after the resignation of the residents can be considered to have the greatest impact due to the government’s policy to increase medical school admissions.

The characteristics of the patients who visited the PED changed. First, the proportions of infants less than 12 months and patients with high acuity increased following resident resignation. Thus, increasing the proportion of pediatric patients requiring emergency care. We were unable to find any studies on the increase in high-acuity pediatric patients after a resident strike. However, a previous South Korean study on adults found a higher proportion of ED visits by high-acuity patients after a resident strike [7]. This is because the decrease in non-emergency patients was more significant compared to the decrease in emergency patients. In general, patients and caregivers think that it is not clear whether the PED of the university hospital is operating normally due to the resignation of residents. For this reason, patients with mild symptoms rather than emergency or critically ill patients further reduced PED visits, and this result was found in this study [11]. Second, the proportion of self-referred patients decreased, whereas the proportion of patients referred from clinics increased. This is thought to have reduced the number of self-referred patients because patients who thought it was unclear whether the PED was operating normally visited clinics first. In addition, it is thought that the proportion of patients referred from clinics increased because emergency patients were included among the patients who visited clinics first [12]. As more pediatric patients visit the clinics, some may require emergency care, resulting in more referrals to the university hospital PED. Third, the proportion of respiratory diseases decreased, and the proportion of neurological, uro-genital, and hemato-oncologic diseases increased. Respiratory disease is one of the most common reasons for PED visits, mostly due to viral infections that are often self-limiting. Therefore, if the pediatric patient did not appear to be in distress, the caregiver would refrain from visiting the PED as much as possible. In contrast, neurological diseases account for relatively few PED visits but are associated with nearly three times as many pediatric intensive care unit admissions as other diseases and a 5.5% PED mortality rate [13, 14]. In our study, epilepsy was the most common primary diagnosis among patients categorized as neurological disorders, followed by migraine and status epilepticus. Hemato-oncological disorders are one of the leading causes of death in children [15], and uro-genital disorders that manifest as symptoms such as hematuria or scrotal pain can be very worrisome for caregivers. These reasons are thought to have contributed to the relative increase in proportions.

The findings of this study are like the changes in PED visit patterns owing to the COVID-19 pandemic. A previous study reported a significant decrease in PED visits by > 50% owing to the COVID-19 pandemic, with an increase in the proportion of infants and high-acuity patients and a decrease in the proportion of PED visits for respiratory diseases [16]. Similar to the changes in PED utilization behavior owing to the COVID-19 pandemic, there has been a decrease in PED use because of increased patient and caregiver anxiety due to residents’ resignation. This demonstrates that the impact of residents’ resignation on patient PED visits was as significant as that of the COVID-19 pandemic. Previous studies reported a small impact of doctor strikes on patient mortality [17]. However, in these studies, the impact may have been limited because the doctors’ strikes lasted only a few days to less than 3 months. Prolonged doctor strikes lasting less than 3 months, such as the current one in South Korea, could eventually lead to adverse effects on mortality and patient outcomes, such as those seen during the COVID-19 pandemic. In addition to the negative impact on patients’ health, the long-term effects of this policy include a decline in the quality of healthcare due to medical education issues and the potential for changes in the global healthcare system due to the outflow of physicians from South Korea. The sudden increase in medical school admission could make it difficult for institutions to provide sufficient quality medical education to medical students, which could result in new doctors who are not adequately trained, which could lead to a long-term decline in quality of care or patient safety issues. There is also the possibility of a brain drain of doctors who rebel against unilateral government policies. This is not only detrimental to the country of origin, but can also affect the healthcare systems of neighboring countries.

### Proposal for returning pediatric residents and improving application rates

The main reasons behind the residents’ resignation were not only the policy to increase the number of doctors but also unresolved issues in the Korean healthcare system, including low reimbursement rates and the high incidence of criminal charges from medical errors [18]. Resolution of these two issues is considered as the prerequisite for the return of the residents. In addition, before implementing any healthcare policy, it is important to have sufficient discussions with various implementing stakeholders, such as doctors, nurses, medical education institutions, and patient organizations, to build trust and take measures to prevent foreseeable problems.

### Limitations

This study has some limitations. First, as a retrospective observational study, it may have been subject to various biases and confounding factors that were not fully controlled. Additionally, this study was conducted at a single tertiary hospital, which may limit the generalizability of the findings to other settings or regions with different patient demographics, hospital resources, and healthcare policies. However, the hospital where the study was conducted is one of Korea’s major university hospitals, and the PED continued to operate without interruption, even after residents’ resignation, making it a reliable representation of the current phenomenon.

## Conclusion

A substantial reduction in both absolute and relative weekly patient visits was observed following the start of the nationwide resident strike at our pediatric emergency department. Additional studies are needed to better understand how this affected pediatric emergency care delivery and access.

## Data Availability

The data that support the findings of this study are available through formal request to the Catholic Open Research Data portal (https://cord.cmcnu.or.kr/). Requests for data access will require approval from the relevant institutional review board. Upon reasonable request and with appropriate permissions, the authors can facilitate access to the analyzed dataset.

## Abbreviations

PED: Pediatric Emergency Department
ITS: Interrupted Time Series
KTAS: Korean Triage and Acuity Scale
GLS: Generalized least squares
ED: Emergency Department

## References

[1] Organization MOHW. A Policy Package to Bring Essential Healthcare Back from the Brink of Collapse Press Release, https://www.mohw.go.kr/board.es?mid=a20401000000%26;bid=0032%26;act=view%26;list_no=1480151%26;tag=%26;nPage=3 (accessed June 11, 2024).

[2] Lee S-J, Cho J-W. Health crisis hits peak warning level as nearly 10,000 trainee doctors resign Korea JoongAng Daily, February 23, 2024, https://koreajoongangdaily.joins.com/news/2024-02-23/national/socialAffairs/Health-crisis-hits-peak-warning-level-as-nearly-10000-trainee-doctors-resign/1987470 (accessed June 11, 2024).

[3] Kim N-y. Trainee doctors of 5 major hospitals to submit resignation en masse by Monday YONHAP NEWS AGENCY, February 16, 2024, https://en.yna.co.kr/view/AEN20240216000451315 (accessed June 11, 2024).

[4] Kang M, Korea Has Left Its Life to Residents… Evidence of a shortage of doctors in 77 hours a week Maeil Business Newpaper(MK), February 22, 2024, https://www.mk.co.kr/en/society/10948996 (accessed June 11, 2024).

[5] Mittiga MR, Schwartz HP, Iyer SB, Rey GD. Pediatric emergency medicine residency experience: requirements versus reality. J Grad Med Educ. 2010;2(4):571–6. 10.4300/JGME-D-10-00106.1. 22132280 10.4300/JGME-D-10-00106.1 PMC3010942

[6] O T, The Moon Jae-in Government’s War Against Young Medical Doctors in South Korea…the Cuba Model? East Asia Research Center, https://eastasiaresearch.org/2020/09/09/moon-jae-ins-war-against-young-medical-doctors-in-south-korea/ (accessed June 20, 2024).

[7] Cho YH, Cho JW, Ryoo HW, Moon S, Kim JH, Lee SH, Jang TC, Lee DE. Impact of an emergency department resident strike during the coronavirus disease 2019 (COVID-19) pandemic in Daegu, South Korea: a retrospective cross-sectional study. J Yeungnam Med Sci. 2022;39(1):31–8. 10.12701/yujm.2021.01130. 34411473 10.12701/yujm.2021.01130 PMC8895968

[8] Lim T, Park J, Je S. Pediatric Korean triage and acuity scale. Pediatr Emerg Med J. 2015;2(2):53–8. 10.22470/pemj.2015.2.2.53.

[9] The 7th. Korean Standard Disease Classification (KCD-7) revision and announcement. [https://kostat.go.kr/board.es?mid=a10301150000%26;bid=246%26;act=view%26;list_no=346904].

[10] Furnivall D, Bottle A, Aylin P. Retrospective analysis of the national impact of industrial action by English junior doctors in 2016. BMJ Open. 2018;8(1):e019319. 10.1136/bmjopen-2017-019319. 29438959 10.1136/bmjopen-2017-019319 PMC5855458

[11] Oh Y, Choi H-R, The irony of the medical crisis… ambulance ‘blind calls’ have decreased THE KOREA ECONOMIC DAILY, Aril 12, 2024, https://www.hankyung.com/article/2024041277731 (accessed August 1, 2024).

[12] Byun E-s. accessed July 21, Parents Who Experienced the ‘ER Roundabout’ Say, ‘We Have No Choice but to Save Our Children on Our Own’ Busan Daily News, May 1, 2024. https://mobile.busan.com/view/busan/view.php?code=2024050118291717564 (2024).

[13] Moreau JF, Fink EL, Hartman ME, Angus DC, Bell MJ, Linde-Zwirble WT, Watson RS. Hospitalizations of children with neurologic disorders in the United States. Pediatr Crit Care Med. 2013;14(8):801–10. 10.1097/PCC.0b013e31828aa71f. 23842588 10.1097/PCC.0b013e31828aa71f PMC3795828

[14] Lopez E, Udaondo J, Olabarri M, Martinez-Indart L, Benito J, Mintegi S. Mortality in Spanish pediatric emergency departments: a 5-year multicenter survey. Eur J Emerg Med. 2017;24(6):392–7. 10.1097/mej.0000000000000365. 26716998 10.1097/MEJ.0000000000000365

[15] Seth R, Singh A. Leukemias in Children. Indian J Pediatr. 2015;82(9):817–24. 10.1007/s12098-015-1695-5. 25680783 10.1007/s12098-015-1695-5

[16] Bae W, Choi A, Kim K, Kang HM, Kim SY, Lee H, Yoo IH, Yang EA, Chun YH, Bin JH, et al. One-year changes in the pediatric emergency department caused by prolonged coronavirus disease 2019 pandemic. Pediatr Int. 2022;64(1):e15016. 10.1111/ped.15016. 34606653 10.1111/ped.15016 PMC8661767

[17] Cunningham SA, Mitchell K, Narayan KM, Yusuf S. Doctors’ strikes and mortality: a review. Soc Sci Med. 2008;67(11):1784–8. 10.1016/j.socscimed.2008.09.044. 18849101 10.1016/j.socscimed.2008.09.044

[18] Yoon JH, Kwon IH, Park HW. The South Korean health-care system in crisis. Lancet. 2024;403(10444):2589. 10.1016/S0140-6736(24)00766-9. 38879246 10.1016/S0140-6736(24)00766-9

